# HTLV-1 Tax-Mediated Inhibition of FOXO3a Activity Is Critical for the Persistence of Terminally Differentiated CD4^+^ T Cells

**DOI:** 10.1371/journal.ppat.1004575

**Published:** 2014-12-18

**Authors:** David Olagnier, Alexandre Sze, Samar Bel Hadj, Cindy Chiang, Courtney Steel, Xiaoying Han, Jean-Pierre Routy, Rongtuan Lin, John Hiscott, Julien van Grevenynghe

**Affiliations:** 1 Lady Davis Institute-Jewish General Hospital, McGill University, Montreal, Quebec, Canada; 2 VGTI Florida, Port St. Lucie, Florida, United States of America; 3 Immunodeficiency Service and Division of Haematology, Royal Victoria Hospital, McGill University Health Center, McGill University, Montreal, Quebec, Canada; University of Pennsylvania School of Medicine, United States of America

## Abstract

The mechanisms involved in the persistence of activated CD4^+^ T lymphocytes following primary human T leukemia/lymphoma virus type 1 (HTLV-1) infection remain unclear. Here, we demonstrate that the HTLV-1 Tax oncoprotein modulates phosphorylation and transcriptional activity of the FOXO3a transcription factor, *via* upstream activation of the AKT pathway. *De novo* HTLV-1 infection of CD4^+^ T cells or direct lentiviral-mediated introduction of Tax led to AKT activation and AKT-dependent inactivation of FOXO3a, *via* phosphorylation of residues Ser253 and Thr32. Inhibition of FOXO3a signalling led to the long-term survival of a population of highly activated, terminally differentiated CD4^+^Tax^+^CD27^neg^CCR7^neg^ T cells that maintained the capacity to disseminate infectious HTLV-1. CD4^+^ T cell persistence was reversed by chemical inhibition of AKT activity, lentiviral-mediated expression of a dominant-negative form of FOXO3a or by specific small interfering RNA (siRNA)-mediated silencing of FOXO3a. Overall this study provides new mechanistic insight into the strategies used by HTLV-1 to increase long-term maintenance of Tax^+^CD4^+^ T lymphocytes during the early stages of HTLV-1 pathogenesis.

## Author Summary

HTLV- infection contributes to the development of Adult T cell Leukemia (ATL) or the neurological disorder HTLV-1-associated myelopathy/tropical spastic paraparesis (HAM/TSP). HTLV-1 principally targets CD4^+^ T lymphocytes and causes profound changes in activation, immune function and cell death. The molecular mechanisms involved in the persistence of infected CD4^+^ T cells following primary HTLV-1 infection remain unclear. We demonstrate here that the Tax oncoprotein inactivates the FOXO3a transcription factor to facilitate the long-term survival of a population of highly activated and terminally differentiated T cells that maintain the capacity to spread infectious viral particles. Mechanistically, expression of Tax oncoprotein in primary human CD4^+^ T cells resulted in the phosphorylation-dependent inactivation of FOXO3a, via the AKT kinase. Tax-mediated CD4^+^ T cell persistence was also reversed by chemical inhibition of the AKT pathway, and reproduced by the expression of a dominant negative version of FOXO3a itself or by silencing its transcriptionally active form using specific siRNA. Overall this study provides new mechanistic insights used by Tax to potentiate the long-term maintenance of CD4^+^ T lymphocytes following HTLV-1 infection and suggests that modulation of FOXO3a activity, using a range of inhibitors targeting the PI3K-AKT-FOXO3a pathway, may offer a valuable addition to current therapeutic approaches.

## Introduction

Infection with the human T cell leukemia virus type I (HTLV-1) affects more than 20 million people worldwide [1] and HTLV-1-associated diseases are a major cause of mortality and morbidity in endemic areas where infection rates range from 2 to 30%. Chronic infection with HTLV-1 can result in a number of severe pathologies, including the aggressive adult T cell leukemia (ATL) and the progressive neurological disorder termed myelopathy/tropical spastic paraperasis (HAM/TSP) [1]. The majority of HTLV-1-infected individuals remain asymptomatic carriers (AC) of the virus but a proportion of AC (1–5%) will develop ATL or HAM/TSP. CD4^+^ T cells are the main targets for viral infection [1], [2], although HTLV-1 can also infect cells of the myeloid lineage including dendritic cells and monocytes [3], [4].

HTLV-1-associated diseases are characterized by profound deregulation of CD4^+^ T cells in terms of activation, immune function and apoptosis [5], [6], all of which are facilitated by the pleiotropic functions of the viral oncoprotein Tax [7]–[10]. In addition to controlling viral gene expression and replication, Tax contributes to malignant transformation of CD4^+^ T cells by modulating host signalling pathways including NF-κB, PI3K-AKT, and JAK-STAT [7]–[10].

The chronic nature of retrovirus infection has been linked to the activity of the Forkhhead box (FOXO) transcription factor family, and particularly to FOXO3a, which can alter the activation, survival and proliferative capacity of CD4^+^ T cell compartment [11]–[15]. FOXO3a is constitutively expressed in most cell types including T lymphocytes, where it regulates apoptosis, tumorigenesis and inflammation [16]–[18], processes that are also deregulated in HTLV-1-associated diseases [5], [19], [20]. Specifically, FOXO3a stimulates expression of pro-apoptotic and anti-proliferative target genes such as *BIM*, *FASL* and *p130* [21]. The FOXO family is subject to numerous post-translational modifications [17] and FOXO phosphorylation can serve either an inhibitory or an activating role in FOXO functions; phosphorylation by JNK activates FOXO3a function [22] while phosphorylation of specific residues (Ser 253 and Thr32) by the serine/threonine kinase AKT inactivates FOXO3a [23].

Previous studies demonstrated that FOXO3a activity contributes to the progressive depletion of central memory CD4^+^ T cells in HIV-1-infected patients [15]. Modulation of FOXO3a activity also occurs during *de novo* HIV-1 infection, where HIV Tat protein induces FOXO3a activity leading to HIV-specific apoptosis [24], [25].

In the present study, we demonstrate that expression of HTLV-1 Tax in primary human CD4^+^ T cells, either by productive HTLV-1 infection or lentiviral-mediated transduction results in the phosphorylation-dependent inactivation of FOXO3a *via* the upstream kinase AKT. FOXO3a inhibition resulted in long-term survival of terminally differentiated, Tax^+^CD27^neg^CCR7^neg^ CD4^+^ T cells that were capable of disseminating infectious HTLV-1. These results provide insight into the mechanisms used by HTLV-1 to increase the long-term maintenance of Tax^+^CD4^+^ T lymphocytes during the early stages of HTLV-1 pathogenesis.

## Results

### Productive HTLV-1 infection is associated with phosphorylation of FOXO3a and persistence of infectious CD4^+^CD27^neg^CCR7^neg^ T cells

**Figure 1:**
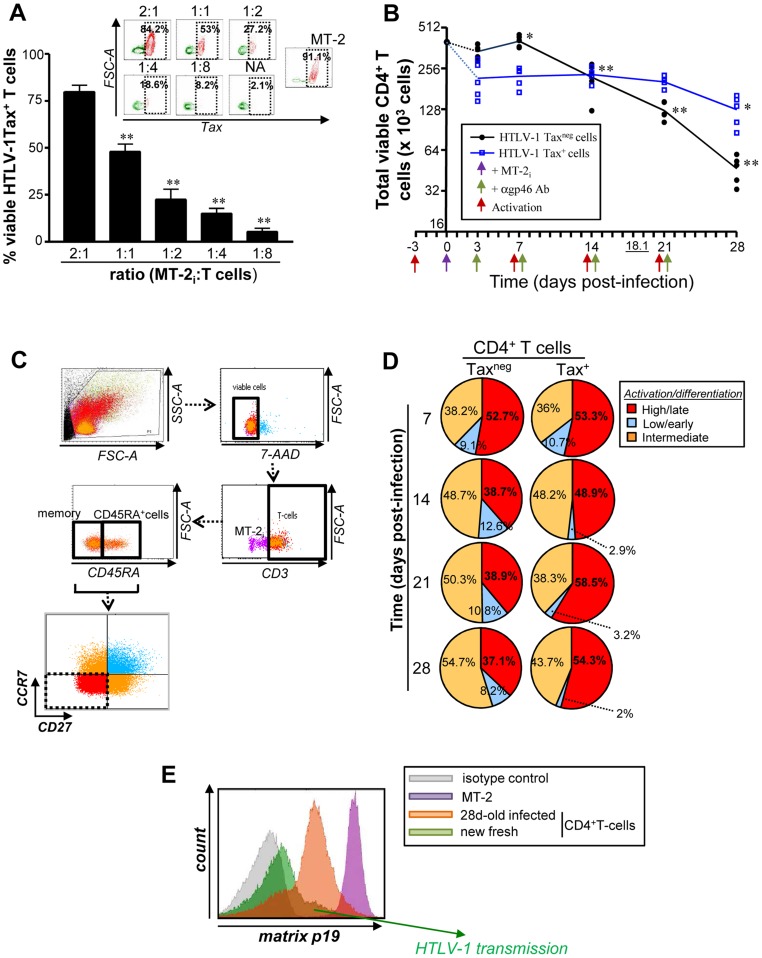
Productive HTLV-1 infection is associated persistence of infectious CD4^+^CD27^neg^CCR7^neg^ T cells. Briefly, activated CD4^+^ T cells were productively infected with MT-2_i_ for 2–28 days to assess their persistence and stepwise differentiation following multiple rounds of TCR stimulation. (A) Tax expression on activated Annexin-V^neg^ CD4^+^ T cells in response to various concentrations of irradiated MT-2 after 48 h pi. We used a CD4/MT-2_i_ ratio of 1∶1 for subsequent experiments. Representative contour plots of five independent experiments were shown above (n = 5). (B) Absolute numbers of total viable Tax^neg^ (black) and Tax^+^ (blue) CD3^+^T cells were determined by trypan blue exclusion. Results are expressed in log_2_ scale. *P* values were determined based on the comparison with cells at 3 dpi. The underlined number represents the half-life of cultured Tax^neg^ T cells. (C) Briefly, cells were collected at 7–28 days pi using long-term co-culture assay and then stained with 7-AAD and anti-CD3-PE, anti-CD45RA-ECD, anti-CD27-APC H7, anti-CCR7-PE Cy7 and anti-Tax-Alexa 647 Abs. Gating strategies to distinguish for three distinct T cell populations, based on their stepwise activation/differentiation status from low/early, intermediate to high/late (CCR7^+^CD27^+^ [blue], single CCR7^+^ or CD27^+^ [orange], CCR7^neg^CD27^neg^cells [red], respectively). (D) Distribution of activated/differentiated 7-AAD^neg^CD3^+^ T cells subsets based on CCR7 and CD27 staining at 7-28 days pi. Pie charts are representative of raw data from five independent experiments. (E) Transmission of HTLV-1 to fresh uninfected PE-stained T cells with 28 day-old HTLV-1-infected CD3^+^ T cells from the same donor (n = 3). Briefly, at 28 day pi infected CD4^+^ T cells were isolated using a CD3 positive selection (StemCell Technology, >97.6% purity). These cells were then co-cultured (ratio 1∶1) for 5 days with activated autologous CD4^+^ T cells that were stained 1 h prior to co-culture with anti-CD3-PE Ab. After 5 days, HTLV-1 transmission was measured on new CD3-PE-stained targets using p19 intracellular staining. MT-2 cells were used as positive control.

Primary CD3/CD28 activated CD4^+^ T cells were infected with HTLV-1 in a dose dependent manner (Fig. 1A) using an *in vitro* trans-infection system in which CD4^+^ T cells were co-cultured with HTLV-1 shedding MT-2 cells [26]. Following multiple rounds of T cell receptor (TCR) triggering, HTLV-1 infected T cells [Tax^+^ cells; blue] persisted for 21–28 days without a significant reduction in cell number, (*P*<0.05) (Fig. 1B); in contrast, T cells that were not infected [Tax^neg^ cells; black] displayed a reduction in cell number by 14 days post-infection (pi) (*P*<0.01). The half-life of gated Tax^neg^CD4^+^ T cells was 18.1 days pi, whereas a half-life calculation could not be determined for Tax^+^ T cells before 28 days.

Using a combination of CD3, CD45RA, CCR7 and CD27 surface markers, we evaluated the generation and maintenance of terminally differentiated (CD3^+^CD45RA^+/−^CCR7^neg^CD27^neg^) T cells during a 28-day cycle of HTLV-1 infection (Fig. 1C) [27], [28]. Repeated TCR triggering reduced the proportion of viable terminally differentiated CD27^neg^CCR7^neg^ effector cells among gated CD3^+^Tax^neg^ T cells, whereas the proportion and cell number of Tax^+^ T lymphocytes increased, and maintained activation status (55.7±4.6% and 36.2±1.3% of CD3^+^CCR7^neg^CD27^neg^cells, respectively for Tax^+^ and Tax^neg^ T cell population at 28 days; *P*<0.01) (Fig. 1D and S1A Fig.). In addition, terminally differentiated Tax^+^CD4^+^ T cells produced infectious HTLV-1, even after four weeks in culture, based on their capacity to transmit virus to freshly isolated autologous CD4^+^ T lymphocytes (Fig. 1E). Overall these results demonstrate that HTLV-1 infection promotes the *in vitro* maintenance of terminally differentiated, virus-producing CD4^+^ T cells (CD3^+^CCR7^neg^CD27^neg^).

**Figure 2:**
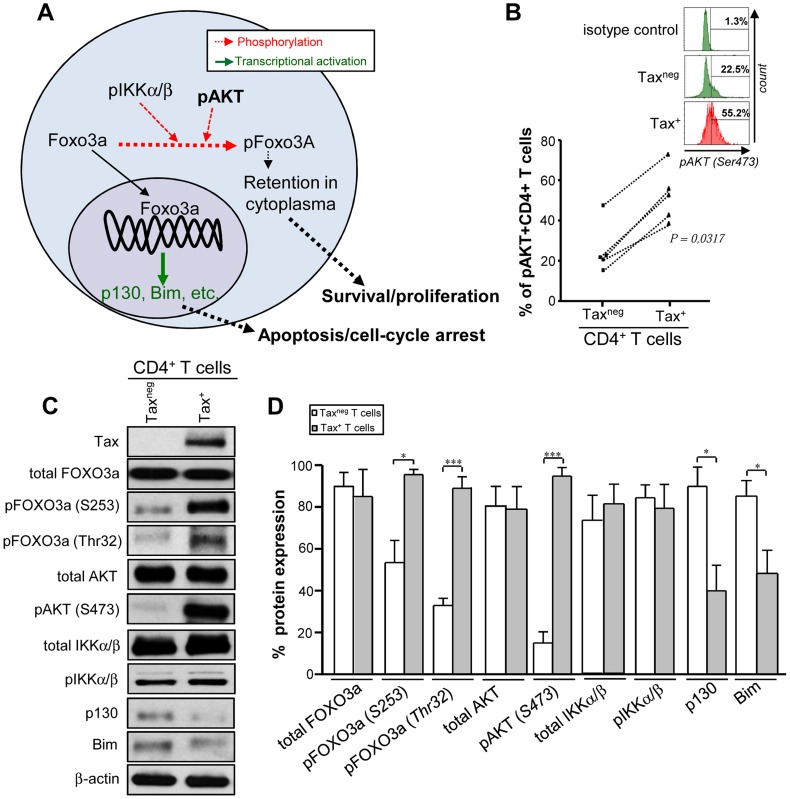
Tax^+^CD4^+^T cells display higher phospho-FOXO3a phenotype than the Tax^neg^subset. (A) Schematic showing the inhibition of FOXO3a signalling pathway by phosphorylation mediated by upstream pro-survival kinases. (B–D) FOXO3a signaling profile on Tax^+^ and Tax^neg^ T cells subsets that are derived from the same culture (in the presence of MT-2i; at ratio 1∶1). (B) PhosFlow analysis of phospho-AKT (Ser473) levels on Tax^+^ and Tax^neg^CD3^+^ T cells at 2 days pi. Representative histograms of pAKT status are also shown above (n = 5). (C) At 2 days pi, 10 µg proteins of highly purified CD3^+^Tax^neg^ and CD3^+^Tax^+^T cell subsets were subjected to immunoblotting to investigate the FOXO3a signalling pathway (n = 3). (D) Densitometric quantification of the spots was performed in parallel using ImageJ software. Results shown represent the mean relative expression ± SD of 3 independent experiments.

We hypothesized that the enhanced cellular survival observed in HTLV-1 infected CD4^+^ T cells may be associated with the deregulation of FOXO3a signalling, given its important role in regulating cell proliferation and apoptosis in other retroviral infections [14], [15], [24], [29]. We first investigated at 2 days pi the activation status of AKT, one of the upstream kinases responsible for phosphorylation of FOXO3a [23] (Fig. 2A). Based on phosphorylation of FOXO3a at Ser473 residue, as detected by PhosFlow and Western Blotting approaches [9], we concluded that the upstream kinase AKT was significantly activated in Tax-expressing cells (*P* = 0.0317 and *P*<0.001, respectively) (Fig. 2B–D). HTLV-1 infection led to an increase in phosphorylation of FOXO3a at residues S253 (*P*<0.01) and Thr32 residues (*P*<0.001) at 2 days pi (Fig. 2C, D), residues that inactivate FOXO3a function [23]. Consistent with this observation, productively infected cells displayed reduced expression of FOXO3a downstream target genes p130 and Bim [15], [23]. The phosphorylation status of IKKα/β, another upstream kinase of FOXO3a, was however unchanged (Fig. 2C, D). Overall these results demonstrate that productive HTLV-1 infection provides a survival and proliferative persistence advantage to infected CD4^+^ T cells, and is associated with AKT-mediated inactivation of FOXO3a transcriptional activity.

### Tax expression inhibits FOXO3a activity *via* activation of AKT

**Figure 3:**
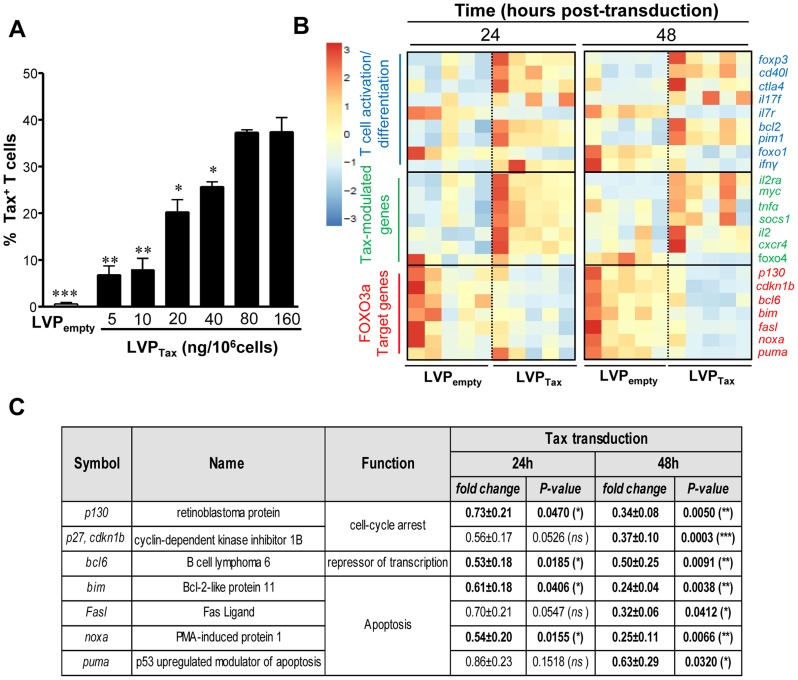
Tax expression inhibits FOXO3a activity *via* activation of AKT. Briefly, activated T cells were transduced by lentiviral particles (LVP) expressing or not Tax. (A) Tax expression at 48 h on transduced T cells in response to LVP_Tax_ concentrations (5–160 ng/10^6^ cells; n = 3). (B) At 24 and 48 h post-transduction, total RNA was extracted, and subjected to Biomark analyses. Heatmaps of genes significantly modulated following Tax expression. (C) Table showing fold changes and *P*-values for several modulated FOXO3a targets genes on Tax-transduced T cells (n = 5; paired *t* test).

**Figure 4:**
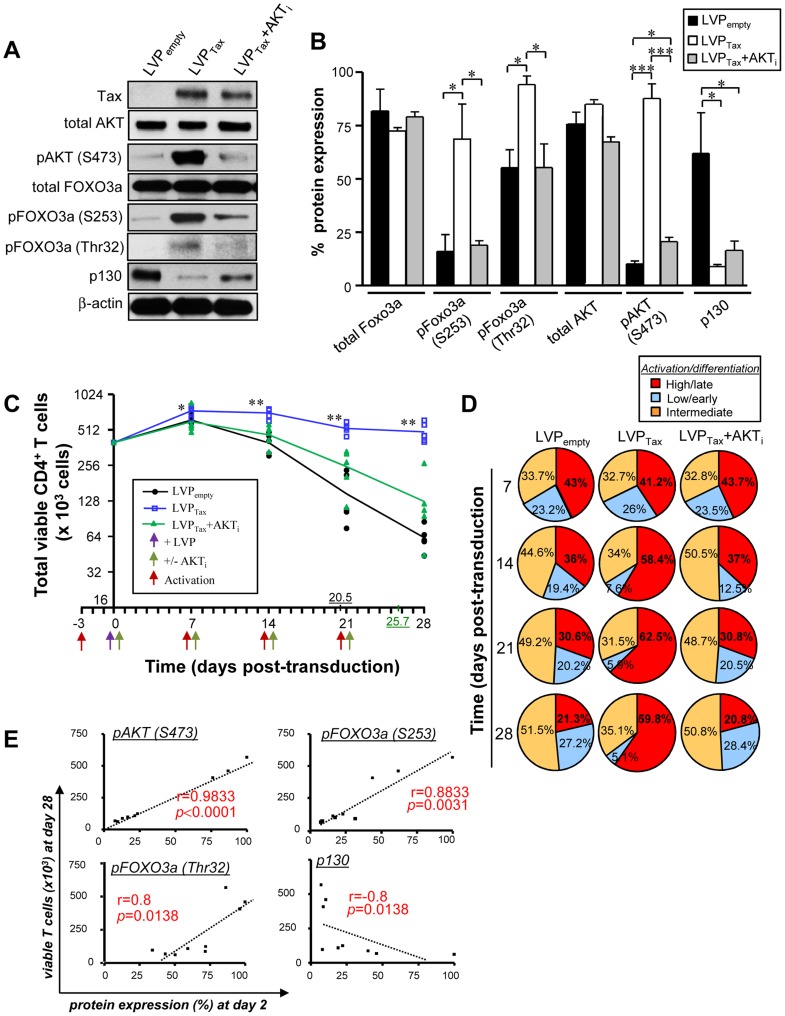
Tax-mediated FOXO3a inactivation is responsible for CD4^+^ T cell persistence. Briefly, activated T cells were transduced by lentiviral particles (LVP) expressing or not Tax for 2–28 days. (A) FOXO3a signaling on transduced T cells expressing or not Tax at 48 h determined by immunoblotting (n = 3). (B) Densitometric quantification of specific bands was performed using ImageJ software. Results shown represent the mean relative expression ± SD of 3 independent experiments. (C–E) Persistence and stepwise differentiation of activated CD4^+^ T cells transduced with LVP expressing Tax in the presence or absence of AKT_i_ (n = 5). (C) Absolute numbers of total viable CD3^+^ T cells were determined by trypan blue exclusion. Results are expressed in log_2_ scale. *P* values were determined based on the comparison with LVP_empty_-transduced cells. The underlined numbers represent the half-life of cultured LVP_empty_ (black) and LVP_Tax_+AKT_i_ (green) conditions. (D) Differentiation status of transduced T cells subsets at 7–28 dpt. Pie charts are representative of raw data from five independent experiments. (E) Correlation between the absolute numbers of viable transduced T cells at 28 days and the levels of expression of FOXO3a-related proteins at 48 h are also shown (n = 9; Spearman test).

Among the proteins encoded by HTLV-1, the Tax oncoprotein exerts its essential role in viral transcription, as well as in T cell transformation [30]–[32]. To determine whether Tax expression alone was sufficient to drive FOXO3a inactivation, HTLV-1 Tax was introduced into activated CD4^+^ T cells using lentiviral particle (LVP)-mediated transduction. Tax was detected using intracellular staining by flow cytometry (Fig. 3A) and a concentration of 80 ng LVP/10^6^cells resulted in ∼40% Tax^+^ cells. LVP_Tax_-transduced CD4^+^ T cells displayed higher expression levels of HTLV-1 Tax when compared to infected cells (S1B Fig.; fold increase ∼2.07; *P* = 0.0091). A BioMark transcriptional high throughput qPCR analysis of LVP-transduced T cells demonstrated that Tax expression modulated mRNA levels of several Tax-modulated genes [8], [31], including an increase in IL-2 and a decrease in type I IFN-associated genes. Tax expression led to higher mRNA expression levels of *CXCR4, SOCS1* and *myc* proto-oncogene, as previously shown [7], [33]–[35]. This analysis also demonstrated the activated/differentiated status of Tax-transduced CD4^+^ T cells, based on the increased expression of *CD40L*, CTLA-4, *IFNγ* and *IL7R* mRNA (Fig. 3B). Interestingly, Tax expression not only inhibited p130 and Bim expression (Fig. 3B, C), but also down-regulated several other FOXO3a target genes; *BCL6*, *p27*, *BIM*, *FASL*, *NOXA* and *PUMA* were all downregulated at the mRNA levels at 24 and 48 h post-transduction (Fig. 3B, C). Tax transduction also induced AKT activation (*P*<0.001) [9], phosphorylation of FOXO3a (*P*<0.05) and inhibition of p130 (*P*<0.05) at the protein level, all of which were significantly reversed by the addition of an AKT inhibitor, AKT inhibitor IV (AKT_i_) (Fig. 4A, B). Conversely, treatment of transduced CD4^+^ T cells with 100 µg/mL IKK inhibitor II (Calbiochem) did not significantly alter the expression levels of phosphorylated FOXO3a (S2B–C Fig.). Tax expression in CD4^+^ T cells did not modulate FOXO3a stability, as we found no significant change in its expression in the presence or absence of Tax even after 6 days of transduction (S3A–B Fig.). Nonetheless Tax transduction resulted in increased nuclear localization of inactive pFOXO3a forms (Ser253 and Thr32 residues) (S3C–D Fig.) [13]. Overall this data demonstrate that Tax expression is sufficient to transcriptionally inactivate FOXO3a signaling, *via* upstream activation of AKT.

### Tax-mediated FOXO3a inactivation is responsible for CD4^+^ T cell persistence

We further investigated whether Tax promoted the persistence of activated CD4^+^ T cells through AKT induction and subsequent FOXO3a inactivation. Tax transduction alone [blue] was sufficient to maintain T cell survival for 28 days, and maintenance of the differentiated T cell population was mediated through AKT signaling, since the addition of the AKT_i_ reduced T cell viability to basal levels (Fig. 4C) (half-lives of transduced T cells with LVP_empty_ [black] and LVP_Tax_+AKT_i_ [green] were 20.5 and 25.7 days, respectively). Tax expression was also associated with the terminally differentiated phenotype, similar to that of HTLV-1 productively infected-T cells (Fig. 4D). A majority of the Tax-transduced CD4^+^ T cells belonged to the CD3^+^CCR7^neg^CD27^neg^subset at 28 days after Tax transduction (55.2±6.3% and 23.9±6.3% for LVP_Tax_ and LVP_empty_ infected T cells, respectively; *P*<0.01). In addition, a strong correlation was established between the number of viable primary CD4^+^ T cells at 28 days post-transduction (Fig. 4C) and the inhibition of FOXO3a signaling observed as early as 2 days pi (Fig. 4A, B), and measured by phosphorylation of AKT (*P*<0.0001), pFOXO3a-S253 (*P* = 0.0031), pFOXO3a-Thr32 (*P* = 0.0138), and expression of p130 (*P* = 0.0138) (Fig. 4E). Altogether, these results demonstrate that long-term survival of activated CD4^+^ T lymphocytes is mediated by a Tax-dependent, AKT phosphorylation and inactivation FOXO3a transcriptional activity.

### Specific inhibition of FOXO3a activity mimics Tax expression

**Figure 5:**
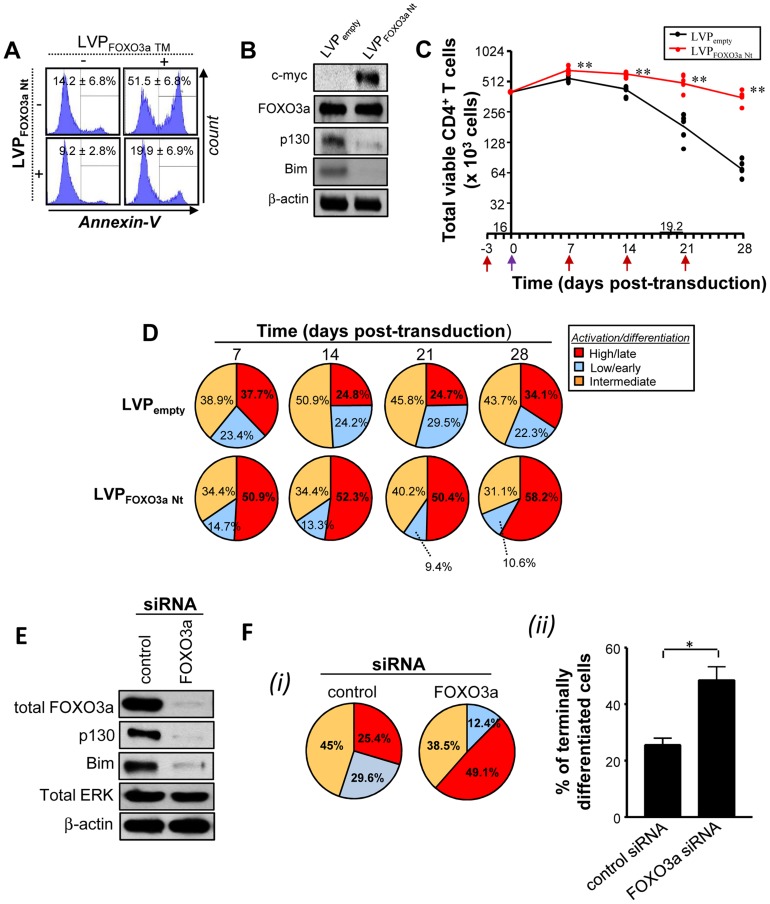
Specific inhibition of FOXO3a activity mimics Tax expression. (A–D) Purified CD4^+^ T cells were pre-activated for 3 days with anti-CD3 and anti-CD28 antibodies and then transduced or not with LVP a dominant-negative form of Foxo3a (Nt) for 2–28 days. (A) Cells were transduced for 48 h with either LVP expressing Foxo3a Nt, or Foxo3a TM (dominant positive), or both. Histograms shown are representative of three independent experiments indicating Annexin-V staining. (B) Immunoblot analyses are performed at 2 days post-transduction to validate the expression of Foxo3a Nt form (c-myc tagged) and subsequent inhibition of endogenous p130 expression (n = 3). (C) Absolute numbers of total viable CD3^+^ T cells were determined by trypan blue exclusion at 7–28 days. *P* values were determined based on the comparison with LVP_empty_-transduced cells. The underlined numbers represent the half-life of cultured LVP_empty_ condition (n = 5). (D) Differentiation status of FOXO3a Nt-transduced T cell subsets at 7–28 days. Pie charts are representative of raw data from five independent experiments. (E, F) Purified CD4^+^ T cells were transfected with control or FOXO3a-specific siRNA and then cultured for two weeks in the presence of TCR triggering. (E) The efficiency of FOXO3a silencing was monitored by immunoblotting after 72 hours of transfection. Western blot analysis of FOXO3a and its targets (p130 and Bim) was performed on transfected cells. The expression of total ERK was also assessed to appreciate the specificity of protein silencing. (F) Differentiation status of FOXO3a siRNA-transfected T cell subsets at 14 days. (*i*) Pie charts are representative of raw data from three independent experiments. (*ii*) % of terminally differentiated CD45RA^+/−^CCR7^neg^CD27^neg^T cells in the presence or absence of FOXO3a silencing are also shown (mean ± SD).

Based on the above results, we rationalized that the T cell persistence observed during HTLV-1 infection or Tax transduction could be reproduced by introduction of a dominant negative form of FOXO3a, termed FOXO3a Nt [15], that encompasses the N-terminal DNA binding domain of FOXO3a (aa1–304) but lacks the C-terminal transactivation domain. FOXO3a Nt acts as a competitive DNA binding inhibitor of transcriptionally active FOXO3a [15], [36] and interferes with FOXO3a activation of pro-apoptotic and anti-proliferative target genes. As shown in Fig. 5A, lentiviral-mediated transduction of FOXO3a Nt prevented primary T cells from undergoing apoptosis (Fig. 5A) and thus mimicked Tax function. Expression of FOXO3a Nt also inhibited endogenous FOXO3a activity, as determined by reduced expression of p130 and Bim (Fig. 5B). FOXO3a Nt expression resulted in persistence of a highly activated, terminally differentiated CD4^+^ T cell population, similar to that observed with Tax expression (Fig. 5C, D). In addition, we found an increased percentage of terminally differentiated CD4^+^ T cells in the presence of specific FOXO3a siRNA at 14 days of culture (Fig. 5E, F and S4 Fig.). Finally, we sought to determine if Tax physically interacted with an inactivated FOXO3a; by co-immunoprecipitation from Tax-transduced primary T cells, we did not observe an interaction between Tax and FOXO3a, although the interaction between Tax and PI3K was detected (S5 Fig.) [9]. It is possible that Tax-PI3K association stimulates AKT activity and thus indirectly contributes to phosphorylation of FOXO3a by AKT. Collectively, these results demonstrate that Tax expression enhanced T cell longevity and activation, through the inhibition of FOXO3a transcriptional activity, mediated by AKT phosphorylation at S253 and Thr32 residues.

## Discussion

HTLV-1 infection is associated with the expansion and leukemic transformation of CD4^+^ T lymphocytes, driven in large part by the chronic disruption of host signaling networks by the HTLV-1 Tax oncoprotein [8], [31], [37], [38]. In the present study, we demonstrate that HTLV-1 infection enhances the *in vitro* cellular persistence of activated CD4^+^ T cells, the expansion of terminally differentiated (CD3^+^CCR7^neg^CD27^neg^) cells and the functional inactivation of the FOXO3a pathway (illustrated by the increased localization of inactive FOXO3a in the nucleus and the inhibition of several targets such as Bim and p130). Mechanistically, both *de novo* HTLV-1 infection and Tax transduction stimulated AKT activation and downstream phosphorylation of FOXO3a at residues S253 and Thr32 (Fig. 2 and 4A, B). Mechanistically, we did not observe an interaction between Tax and FOXO3a (S5 Fig.), although the interactions between Tax and PI3K was detected as previously reported [9]. It is possible that Tax-PI3K association stimulates AKT activity and thus indirectly contributes to phosphorylation of FOXO3a by AKT. Also, we cannot exclude the possibility that post-translational modifications other than phosphorylation (such as acetylation, methylation, ubiquitination) may impact FOXO3a activity [36], [39]. In addition, since mRNA and protein levels of Tax are generally barely detectable in ATL cells displaying constitutively active AKT [40], [41], it is possible that other Tax-independent mechanisms of FOXO3a inactivation may be used by HTLV-1. Nevertheless, Tax-transduced T cells displayed a global inhibition of FOXO3a activity, illustrated by reduced expression of many pro-apoptotic and anti-proliferative target genes such as *BIM, FASL, NOXA, p27 and p130* at 24–48 h post-transduction (Fig. 3B, C). Overall this study provides new mechanistic insights by which Tax potentiates the long-term maintenance of CD4^+^ T lymphocytes following HTLV-1 infection.

FOXO3a activity is also targeted by another HTLV-1 accessory protein, HBZ, which was shown to inhibit FOXO3a by interfering with its localization and ability to bind DNA [13]. The mechanistically distinct, yet functionally redundant, inhibition of FOXO3a signaling may be explained by the distinct kinetics of expression of these two regulatory proteins. It has been reported that, in contrast to Tax, HBZ is transcribed at high levels in chronically infected patient samples [42]. Conversely, even though Tax mRNA expression is relatively moderate, it is at its highest during the early stages of infection, specifically within the first week [43]. Additionally, Tax controls FOXO4 activity through degradation by the proteasome during ATL development [11]. The inhibition of FOXO3a or FOXO4 activity by distinct HTLV-1 accessory mechanisms also highlights the importance of FOXO inactivation as a strategy to perpetuate HTLV-1 infected CD4^+^ T lymphocytes and to contribute in the ATL development.

Using a BioMark high throughput qPCR analysis (S1 Table), we demonstrated that Tax not only mediated CD4^+^ T cell persistence through the inactivation of the FOXO3a pathway, but also down regulated type I IFN responses (S6A Fig.), in part mediated by the negative regulator of the JAK-STAT1 pathway SOCS1 [7], [34]. Taken together with our findings, these data indicate an involvement of Tax oncoprotein in targeting FOXO3a to concomitantly modulate the cell survival, as well as the type I IFN antiviral responses in CD4^+^ T cells and thus facilitate HTLV-1 infection.

The identification of a pivotal role for FOXO3a in *de novo* HTLV-1 infection of CD4^+^ T cells in terms of cellular differentiation and persistence survival may have important consequences for retroviral pathogenesis. For instance, alterations in the microenvironment mediated by HIV infection significantly increase FOXO3a activity, with a major impact on T and B cell immunity and survival [14], [15], [44], [45]. Kino *et al*. reported that the HIV accessory protein Vpr inhibited the ability of insulin to induce FOXO3a phosphorylation via AKT, thus interfering with its exclusion from the nucleus [46]. The expression of HIV-1 regulatory molecule Tat in specific T cells and macrophages also induced FOXO3a-mediated apoptosis [24], [47]. FOXO3a activity also impacts the pathogenesis and the outcome of Abelson murine leukemia virus [29].

Several gene networks/pathways that were deregulated in Tax expressing CD4^+^ T cells were similarly disrupted in transcriptome analyses of PBMC from HTLV-1 infected individuals [34], [48], [49] (S5B Fig.). For instance, transcriptional analysis of differentially regulated pathways demonstrated that cytokines IL15, IL17R, IL7R were down regulated, and chemokine CXCR4 was up regulated in ATL patients; in contrast TNFRSF17 was down regulated while granzyme B and IL-2 were up regulated, in HAM/TSP and AC individuals (S5B Fig.). It is tempting to speculate that disruption of signalling mechanisms identified early after *de novo* HTLV-1 infection are also important in the development and maintenance of HTLV-1 associated pathologies and could be targeted for clinical treatment.

Due to poor prognosis of patients diagnosed with ATL, coupled with limited therapeutic options, novel immunological approaches including recombinant IL-7, IFN-α, and neutralizing anti-CD25 or anti-CXCR4 antibodies are currently being used to treat ATL and HAM/TSP patients [50]–[53]. The present study indicates that the PI3K-AKT-FOXO3a pathway may also represent a potential therapeutic target in ATL patients. Since AKT inhibitors are already in clinical development [54], they may offer a valuable addition to current therapeutic approaches.

## Materials and Methods

### Products

RPMI-1640 media, FBS and antibiotics were provided by Wisent Technologies (CA, USA). Unconjugated anti-Tax mAbs (clone LT4) was generously provided by Dr. Yuetsu Tanaka (Kitasato University, Kanagawa, Japan). MT-2 cell lines were obtained from the ATCC (VA, USA). All antibodies used for flow cytometry were purchased from BD Biosciences, except for the antibody to CD45RA-ECD, which was from Beckman Coulter. All primary antibodies used in Western Blots (anti-phospho forms of FOXO3a, anti-Bim, anti-ERK, anti-AKT, anti-PI3K p85, anti-IKK, and anti-phospho-IKK Abs) were purchased from Cell Signaling Technology Inc., whereas anti-p130 and anti-actin were purchased from Sigma Aldrich; anti-FOXO3a from Abcam. 7-Aminoactinomycin D (7-AAD) came from Invitrogen. Anti-Tax antibody (clone 1A3) was purchased from Santa Cruz Biotechnology.

### Ethics statement

Leukaphereses from healthy donors were obtained from the Royal Victoria Hospital, Montreal (QC, Canada). Written informed consent approved by the Royal Victoria Hospital and the Jewish General Hospital review boards was provided to study participants. Research was conformed to ethical guidelines established by the ethics committee of the Royal Victoria Hospital, the Jewish General Hospital, and McGill University (# BMB-2001-028).

### Purification and activation of CD4^+^ T cells

PBMCs were isolated using Ficoll-Hypaque gradient and CD4^+^ T cells were then purified using the untouched CD4 isolation kit (EasySep Human CD4^+^ T cell Enrichment Kit; StemCell Technologies, Vancouver, BC, Canada), allowing for more than 94% purification without any cell stimulation and apoptosis. CD4^+^ T cells were then activated 72 hours in RPMI complete in the presence of 1 µg/mL anti-CD28 (BD Biosciences) in 6 well plates pre-coated 24 hours earlier with 0.5 µg/mL anti-CD3 (clone: OKT-3, BioLegend; 2.10^6^ cells/well).

### HTLV-1 trans-infection

Cell-cell transmission of HTLV-1 was performed essentially as previously described [26]. 20.10^6^ HTLV-1 produced cell line MT-2 were first irradiated at 15,000 rads and then mixed at various ratios (2∶1 to 1∶8) of irradiated MT-2 to activated CD4^+^ T cells. At several time points post-infection (pi), collected cells were treated with Cell Dissociation Solution non-enzymatic according to the Sigma manufacturer's protocol and finally filtered (70 µm), prior further analyses.

### Flow cytometry

#### Surface staining

At day 2 pi, cells were first stained in calcium buffer with anti-CD3-PE Cy7 antibody (Ab) and Annexin-V-V450 in the presence or absence of anti-CD25-APC Cy7 or anti-HLA DR-Alexa700 Abs for 10 minutes at 4°C.

#### HTLV-1 Tax quantitation

Cells were washed twice and fixed at room temperature (RT) in BD FACS Lysing Buffer (Becton Dickinson) and incubated with anti-Tax-FITC and anti-p19-Alexa647 mAbs for 20 minutes at RT in 0.25% saponin. Anti-p19 IgG_1_ antibody was conjugated to Alexa647 dye using the Zenon mouse IgG_1_ labeling kit (number: Z25008; Life Technologies Inc., ON, USA) according to the manufacturer's protocol.

#### pAKT measurement

AKT phosphorylation was measured using BD Bioscience PhosFlow anti-pAKT (S473 residue) specific Ab, as previously described [23].

### Western blotting and Tax co-immunoprecipitation (co-IP)

Protein lysates (2–10 µg) from highly purified CD4^+^ T cell subsets were subjected to Western blot analysis as previously described [4]. Densitometric quantifications of protein of interest (normalized to β-actin whose expression level was used as loading control) were calculated using ImageJ software.

#### Anti-Tax co-IP

MT-2 cells were collected to determine molecular interactions with HTLV-1 Tax. Briefly, cells were lysed using CHAPS buffer with protease inhibitors as previously described [4]. 500 µg of proteins were used for this assay. Monoclonal antibodies (clones 1A3 and LT4) were used to immunoprecipitate and immunoblot for Tax respectively. Proteins (20 µg) were also collected and referred to as the "input" fraction.

### Generation of recombinant lentiviruses

The lentiviral vector pWPI (empty vector), packaging plasmid psPAX2 and envelope plasmid pMD2G were generously provided by VGTI-Florida, whereas pCLXSN-Tax vector was purchased from Addgene (ref: 44038; MA, USA). The FOXO3a N-terminal (Nt) fragment was cloned into pWPI, and as previously described [15], lentiviral particles were produced in 293T cells that harboured either pWPI or pCLXSN (empty controls), pWPI-FOXO3a Nt, or pCLXSN-Tax expression vector. Titers (ng/mL) of lentiviral constructs were assessed using HIV p24 ELISA (Zeptometrix Corporation, USA).

### Long-term persistence assays

CD4^+^ T cells (4.10^4^) were co-cultured in the presence of irradiated MT-2 cells (ratio  = 1∶1) in complete RPMI. At 3 days pi, cells were washed to remove the maximum of dead MT-2_i_ and cultured in the presence of 1 µg/mL anti-gp46 Abs to avoid any de novo infection. On days 7, 14 and 21 pi, cells were re-stimulated by adding fresh anti-CD3 and anti-CD28 Ab in the presence of anti-gp46 Abs (at 10 µg/mL; Abcam) to avoid any de novo infection. The efficiency of neutralizing anti-gp46 Ab was confirmed by the absence of p19 staining on primary cells that were treated since the onset of co-culture. At days 3, 7, 14, 21 and 28 pi, viable cultured cells were counted by trypan blue exclusion and stained with 7-AAD, anti-CD3-PE, anti-CD45RA-ECD, anti-CD27-APC H7, anti-CCR7-PE Cy7 and anti-Tax-Alexa 647 Ab for flow cytometry analyses.

### FOXO3a silencing

A total of 10^7^ activated CD4^+^ T cells were first electroporated in the presence of control or FOXO3a-specific siRNA (Invitrogen; 3 µg) using nucleofector II technology, according the manufacturer's protocol (Amaxa human T cell nucleofector kit). Transfected cells were then washed, and cultured alone for 14 days as described above in Long-term persistence assays.

### Fluidigm BioMark assays

Total RNA was isolated from cells using RNeasy Kit (Qiagen, Valencia, USA) as per manufacturer's instructions. RNA was reverse transcribed using the SuperScript VILO cDNA synthesis kit according to manufacturer's instructions (Invitrogen, Carlsbad, USA). PCR primers were designed using Roche's Universal Probe Library Assay Design Center (www.universalprobelibrary.com) and ordered from the Integrated DNA Technology company (IDT, USA) (S1 Table). cDNA along with the entire pool of primers were pre-amplified for 14 cycles using TaqMan PreAmp Master Mix as per manufacturer's protocol (Applied Biosystems, Foster City, USA). cDNA were exonuclease treated to get rid of excess primers using Exonuclease I (*E. coli*) (New England Biolabs, Ipswich, USA). cDNA samples were prepared with 2× FastStart TaqMan Probe Master (Roche, Penzberg, Germany), GE sample loading buffer (Fluidigm, San Francisco, USA) and Taq Polymerase (Invitrogen, NY, USA). Assays were prepared with 2× assay loading reagent (Fluidigm, NY, USA), primers (IDT) and probes (Roche, Penzberg, Germany). Samples and assays were loaded in their appropriate inlets on a 48.48 BioMark chip. Chip was run on the Biomark HD System (Fluidigm, San Francisco USA) and enabled quantitative measurement of up to 48 different mRNAs in 48 samples under identical reaction conditions. Raw Ct values were calculated after 40 cycles by the real time PCR analysis software (Fluidigm, San Francisco, USA) and software designated failed reactions were discarded from analysis. All data are presented as a relative quantification with efficiency correction based on the relative expression of target gene versus the mean of (*gapdh*+*actin*+*β2 microglobulin*) as the invariant control. The N-fold differential expression of mRNA gene samples was expressed as 2^ΔΔCt^. The heatmap was produced with the R package pheatmap (http://CRAN.R-project.org/package=pheatmap) and gene expression is shown as gene-wise standardized expression (Z score).

### Statistical analysis

**S**tatistical analyses were performed as previously described [4]. ***, *P*<0.001; **, *P*<0.01 and *, *P*<0.05.

## Supporting Information

S1 Fig ***In vitro*** **assay of primary T cells (trans-infection with MT-2_i_ cells and transduction with LVP_Tax_).** (A) Activated CD4^+^ T cells were productively infected with MT-2_i_ for 7–28 days to assess the proportion of HTLV-1 infected T cells during the time course of the co-culture. Results shown represent the percentages of Annexin-V^neg^Tax^+^CD3^+^ T cells at each time points (n = 3) (B) Comparison of Tax expression levels between productively infected (n = 5) and Tax-tranduced (n = 3) CD4^+^ T cells at 48 hours. The percent of Tax^+^CD4^+^ T cells (black) and the Tax expression levels on positive cells (red) are shown.
(TIF)

S2 Fig **Inhibition of IKK does not impact the phosphorylation of FOXO3a.** (A, B) Briefly, activated CD4^+^ T cells were transduced for 48 h with LVP_Tax_ in the presence or absence of IKK inhibitor II. (B) Representative blots at 48 h post-transduction are shown for pFOXO3a and pIKK expression profiles. (C) Densitometric quantification of three independent experiments was performed using ImageJ software (mean ± SD).
(TIF)

S3 Fig **Tax expression does not alter the expression of total FOXO3a, but results in increased nuclear localization of inactive phosphorylated forms.** (A, B) Purified CD4^+^ T cells were transduced with LVP_empty_ or LVP_Tax_ for 2 to 6 days and collected at each time points. (A) Western blot analysis of total FOXO3a expression was performed on transduced cells until 6 days. (B) Densitometric quantification of specific bands was performed using ImageJ software (n = 3). (C, D) After two days of transduction, CD4^+^ T cells were collected and subjected to nuclear extraction as previously performed [4]. (C) Western blots performed on purified nuclear fractions in the presence or absence of Tax expression. Purity of nuclear fractions was determined using antibodies against nuclear (Histone_H3_) and cytosolic (COX-IV) proteins. (D) Densitometric quantification of three independent experiments for the expression levels of pFOXO3a forms in the nuclear fractions (mean ± SD).
(TIF)

S4 Fig **Silencing FOXO3a expression using small interfering RNA (siRNA) increased the number of CD4^+^ T cells (n = 3).** (A, B) Purified CD4^+^ T cells were transfected with control or FOXO3a-specific siRNA and then cultured for two weeks in the presence of TCR triggering. (A) The efficiency of FOXO3a silencing was monitored by immunoblotting after 72 hours of transfection using Western Blotting. Results shown are the densitometric analysis of bands using ImageJ software. (B) At day 14 of culture, transfected CD4^+^ T cells were also collected to assess numbers of total viable CD4^+^ T cells that were determined by trypan blue exclusion. *P* values were determined based on the comparison with LVP_empty_-transduced cells.
(TIF)

S5 Fig **HTLV-1 Tax interacts with PI3K but not FOXO3a (n = 4).** Primary CD4^+^ T cells were transduced for 48 h with LVP_Tax_, then collected and lysed using CHAPS buffer to assess (A) anti-Tax co-immunoprecipitation (co-IP). Representative blots from two separate experiments are shown, including (B) the "input" fractions. We also included lysates that were immunoprecipitated with an isotype IgG1 (instead of anti-Tax antibody) as negative control to help differentiate non-specific background signal from specific antibody signal.
(TIF)

S6 Fig **Transcriptome analysis of HTLV-1 Tax expressing CD4^+^ T cells.** (A) Briefly, activated T cells transduced or not with LVP_Tax_ were collected at 24 and 48 h post-transduction and subjected to Biomark analysis (n = 5). Heatmap analysis of genes (not shown in Fig. 3B) that were significantly modulated following Tax expression. (B) Table listing several genes that were dysregulated during both acute Tax transduction (blue) and in chronically infected individuals (red). List includes genes which are currently involved as therapeutics in ATL and HAM/TSP patients.
(TIF)

S1 Table **List of primers used for the Biomark analyses.** This list includes sequences and appropriate gene nomenclature.
(TIF)
