# Supplementary figures and images for: HTLV-1 Tax-Mediated Inhibition of FOXO3a Activity Is Critical for the Persistence of Terminally Differentiated CD4+ T Cells
Source: PLoS Pathog. 2014 Dec 18;10(12):e1004575. doi: 10.1371/journal.ppat.1004575 (PMC4270795; doi:10.1371/journal.ppat.1004575)

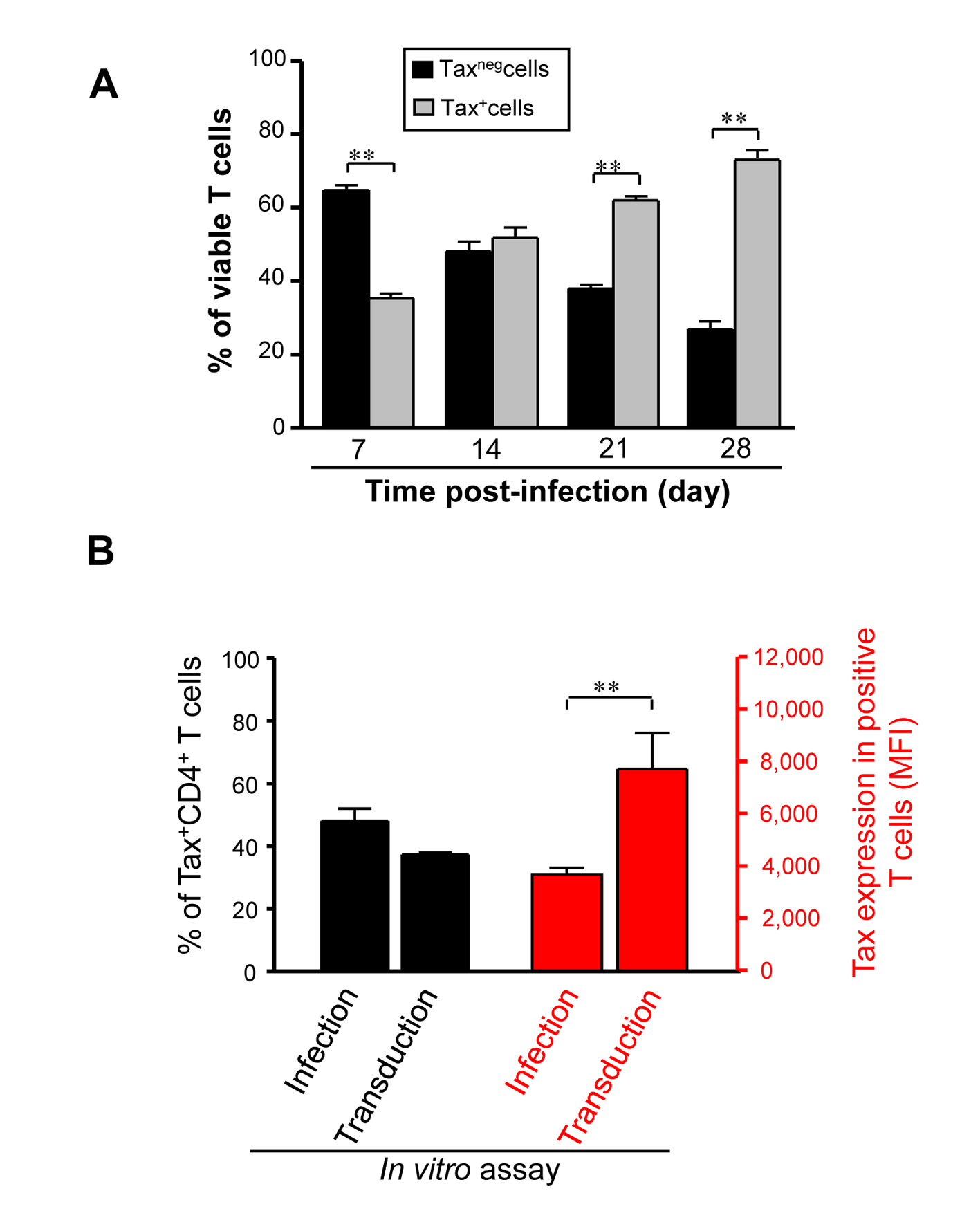

Supplement: S1 Fig — In vitro assay of primary T cells (trans-infection with MT-2i cells and transduction with LVPTax). (A) Activated CD4+ T cells were productively infected with MT-2i for 7–28 days to assess the proportion of HTLV-1 infected T cells during the time course of the co-culture. Results shown represent the percentages of Annexin-VnegTax+CD3+ T cells at each time points (n = 3) (B) Comparison of Tax expression levels between productively infected (n = 5) and Tax-tranduced (n = 3) CD4+ T cells at 48 hours. The percent of Tax+CD4+ T cells (black) and the Tax expression levels on positive cells (red) are shown. (TIF) [file ppat.1004575.s001.tif]

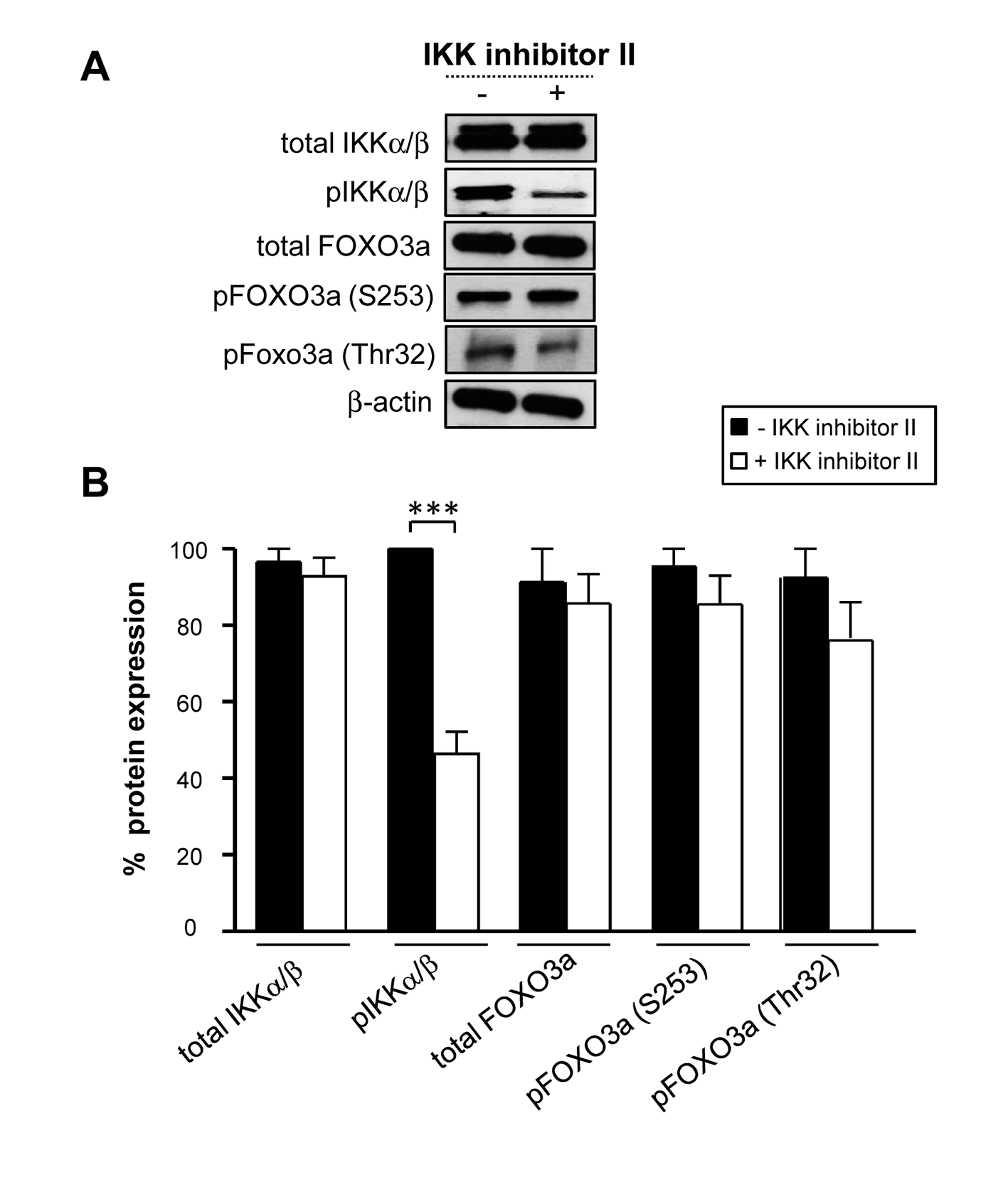

Supplement: S2 Fig — Inhibition of IKK does not impact the phosphorylation of FOXO3a. (A, B) Briefly, activated CD4+ T cells were transduced for 48 h with LVPTax in the presence or absence of IKK inhibitor II. (B) Representative blots at 48 h post-transduction are shown for pFOXO3a and pIKK expression profiles. (C) Densitometric quantification of three independent experiments was performed using ImageJ software (mean ± SD). (TIF) [file ppat.1004575.s002.tif]

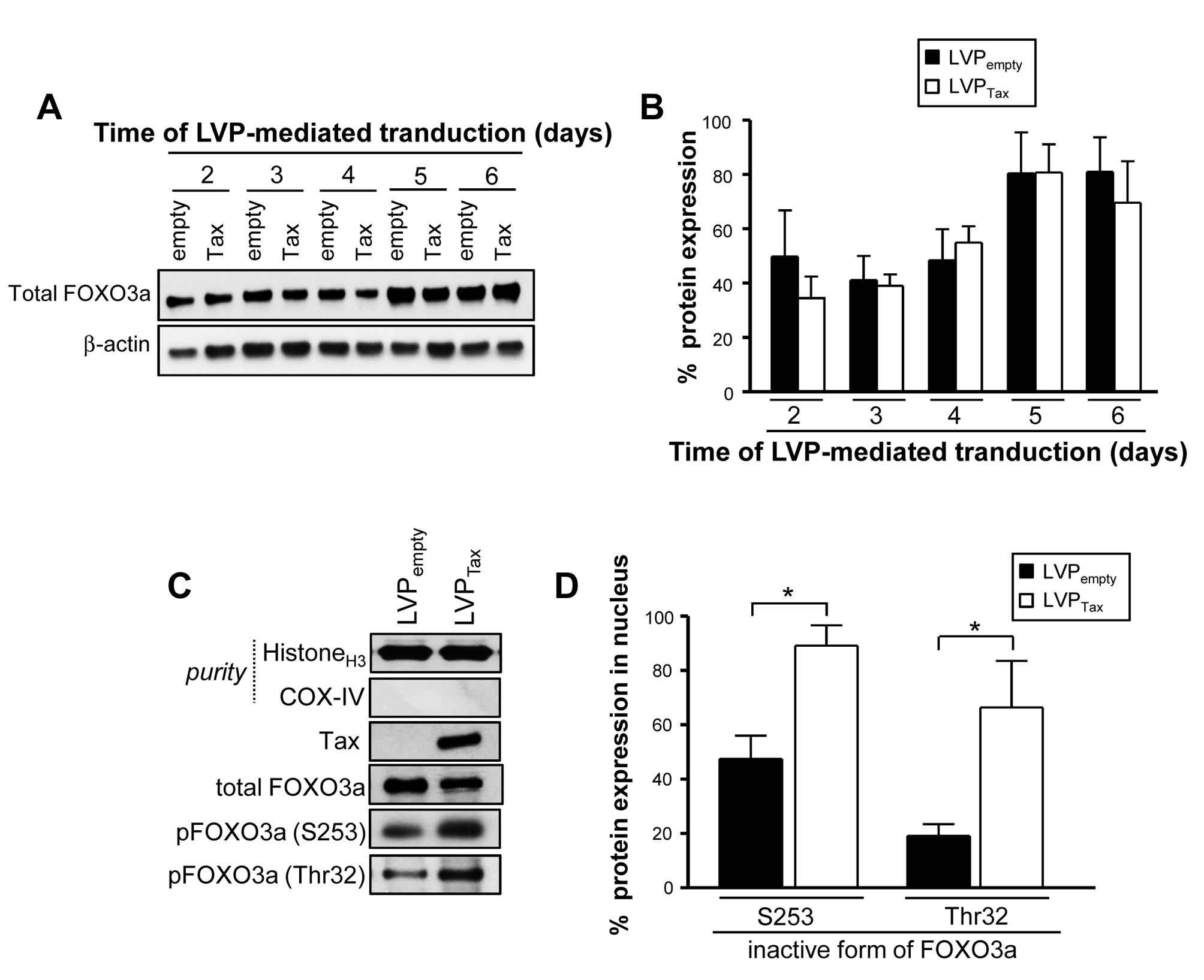

Supplement: S3 Fig — Tax expression does not alter the expression of total FOXO3a, but results in increased nuclear localization of inactive phosphorylated forms. (A, B) Purified CD4+ T cells were transduced with LVPempty or LVPTax for 2 to 6 days and collected at each time points. (A) Western blot analysis of total FOXO3a expression was performed on transduced cells until 6 days. (B) Densitometric quantification of specific bands was performed using ImageJ software (n = 3). (C, D) After two days of transduction, CD4+ T cells were collected and subjected to nuclear extraction as previously performed [4]. (C) Western blots performed on purified nuclear fractions in the presence or absence of Tax expression. Purity of nuclear fractions was determined using antibodies against nuclear (HistoneH3) and cytosolic (COX-IV) proteins. (D) Densitometric quantification of three independent experiments for the expression levels of pFOXO3a forms in the nuclear fractions (mean ± SD). (TIF) [file ppat.1004575.s003.tif]

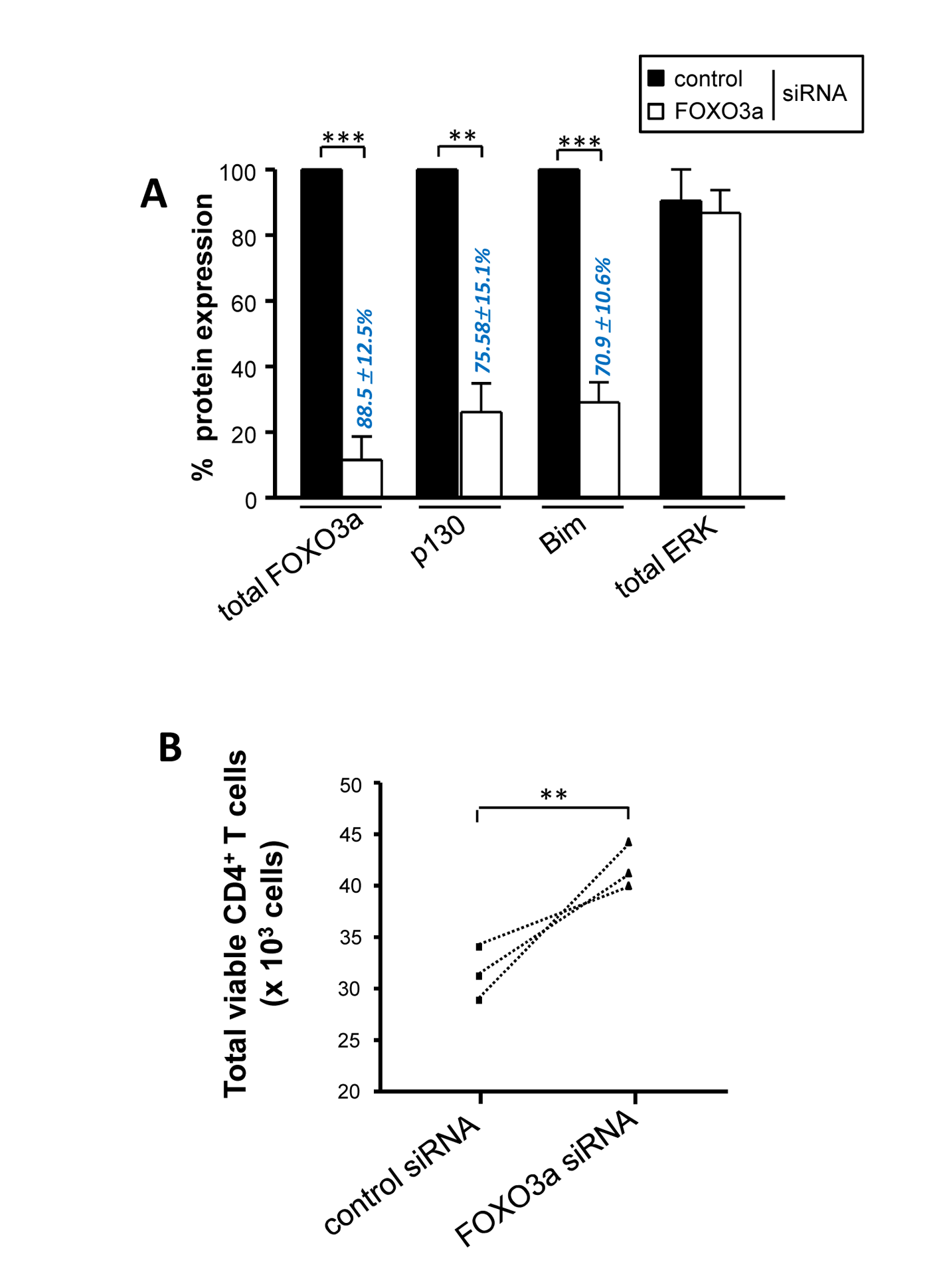

Supplement: S4 Fig — Silencing FOXO3a expression using small interfering RNA (siRNA) increased the number of CD4+ T cells (n = 3). (A, B) Purified CD4+ T cells were transfected with control or FOXO3a-specific siRNA and then cultured for two weeks in the presence of TCR triggering. (A) The efficiency of FOXO3a silencing was monitored by immunoblotting after 72 hours of transfection using Western Blotting. Results shown are the densitometric analysis of bands using ImageJ software. (B) At day 14 of culture, transfected CD4+ T cells were also collected to assess numbers of total viable CD4+ T cells that were determined by trypan blue exclusion. P values were determined based on the comparison with LVPempty-transduced cells. (TIF) [file ppat.1004575.s004.tif]

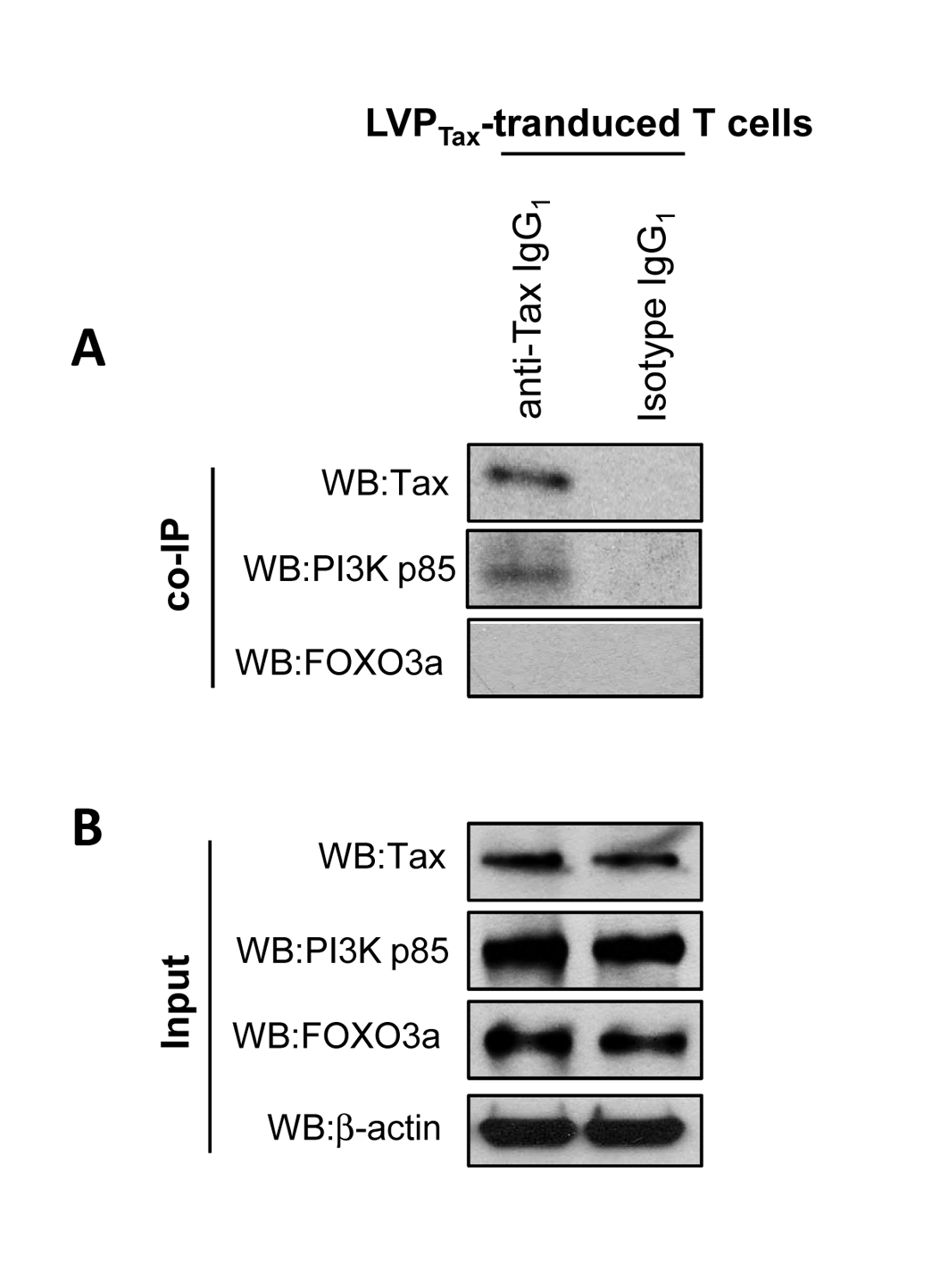

Supplement: S5 Fig — HTLV-1 Tax interacts with PI3K but not FOXO3a (n = 4). Primary CD4+ T cells were transduced for 48 h with LVPTax, then collected and lysed using CHAPS buffer to assess (A) anti-Tax co-immunoprecipitation (co-IP). Representative blots from two separate experiments are shown, including (B) the "input" fractions. We also included lysates that were immunoprecipitated with an isotype IgG1 (instead of anti-Tax antibody) as negative control to help differentiate non-specific background signal from specific antibody signal. (TIF) [file ppat.1004575.s005.tif]

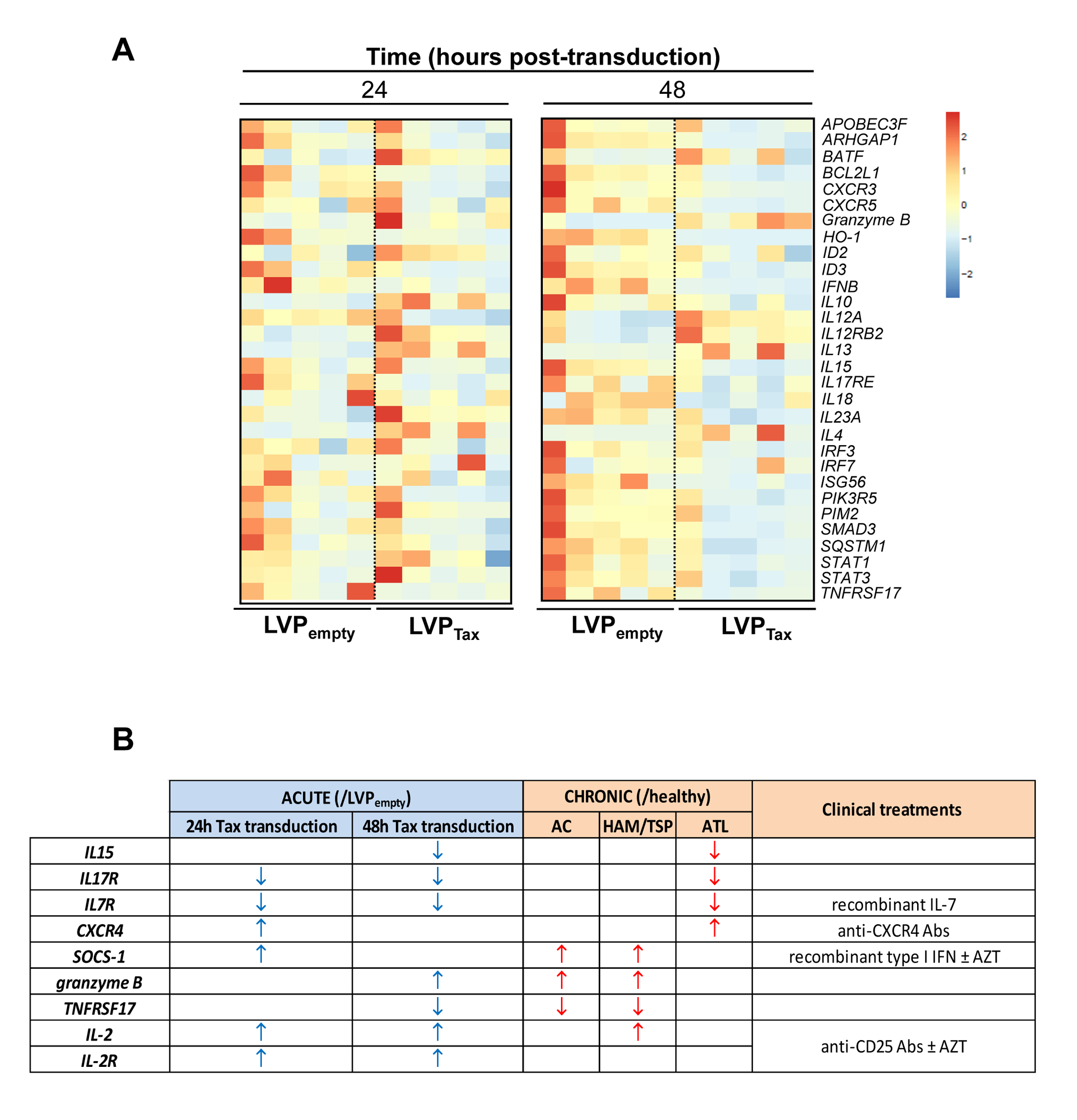

Supplement: S6 Fig — Transcriptome analysis of HTLV-1 Tax expressing CD4+ T cells. (A) Briefly, activated T cells transduced or not with LVPTax were collected at 24 and 48 h post-transduction and subjected to Biomark analysis (n = 5). Heatmap analysis of genes (not shown in Fig. 3B) that were significantly modulated following Tax expression. (B) Table listing several genes that were dysregulated during both acute Tax transduction (blue) and in chronically infected individuals (red). List includes genes which are currently involved as therapeutics in ATL and HAM/TSP patients. (TIF) [file ppat.1004575.s006.tif]

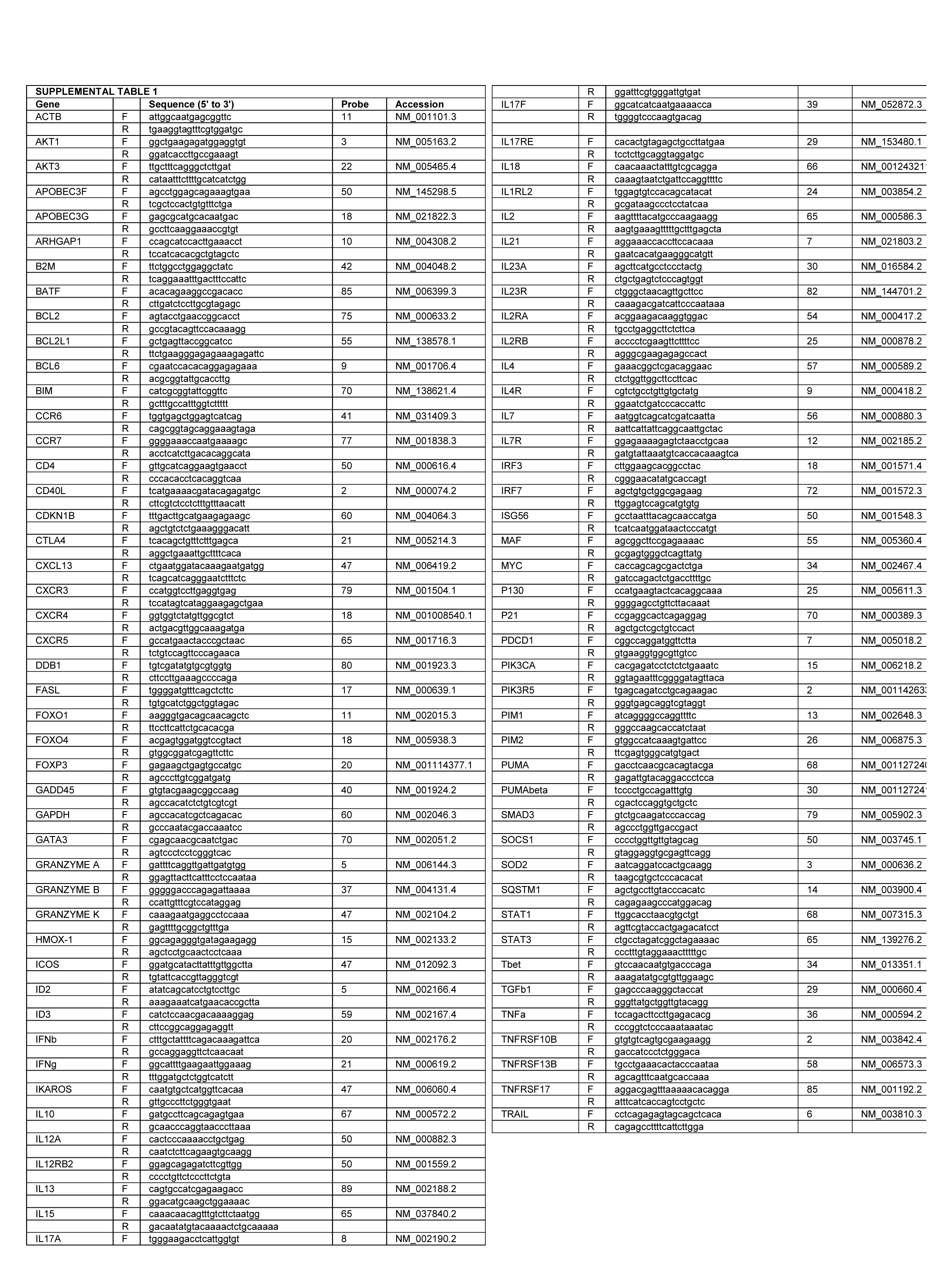

Supplement: S1 Table — List of primers used for the Biomark analyses. This list includes sequences and appropriate gene nomenclature. (TIF) [file ppat.1004575.s007.tif]
